# *catena*-Poly[[[dibromidomanganese(II)]-μ-2,2′-bipyrimidine-κ^4^ *N* ^1^,*N* ^1′^:*N* ^3^,*N* ^3′^]dihydrate]

**DOI:** 10.1107/S1600536811049919

**Published:** 2011-11-30

**Authors:** Kwang Ha

**Affiliations:** a School of Applied Chemical Engineering, The Research Institute of Catalysis, Chonnam National University, Gwangju 500-757, Republic of Korea

## Abstract

The asymmetric unit of the title compound, {[MnBr_2_(C_8_H_6_N_4_)]·2H_2_O}_*n*_, contains one half of a repeat unit of the neutral linear coordination polymer and a solvent water mol­ecule, with the Mn^II^ ion on a crystallographic twofold axis. In the polymer, inversion-related Mn^II^ ions are bridged by the bis-chelating 2,2′-bipyrimidine (bpym) ligands, thereby forming a chain structure along the *c*-axis direction, and are six-coordinated in a distorted *cis*-N_4_Br_2_ octa­hedral environment by four N atoms of twofold-related bpym ligands and twofold-related bromide anions. In the crystal, the complex polymers and solvent water mol­ecules are linked by inter­molecular O—H⋯Br and C—H⋯O hydrogen bonds, forming a two-dimensional layered structure extending parallel to the *ac* plane.

## Related literature

For the crystal structure of the chlorido Mn^II^ complex polymer [MnCl_2_(bpym)]_*n*_.2*n*H_2_O, which is isotypic to the title compound, see: Armentano *et al.* (2003 ▶).
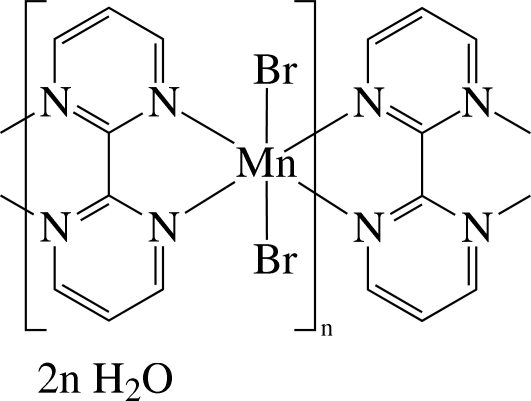


## Experimental

### 

#### Crystal data

[MnBr_2_(C_8_H_6_N_4_)]·2H_2_O
*M* *_r_* = 408.96
Monoclinic, 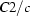

*a* = 17.950 (9) Å
*b* = 8.263 (4) Å
*c* = 10.188 (5) Å
β = 123.888 (8)°
*V* = 1254.4 (10) Å^3^
*Z* = 4
Mo *K*α radiation
μ = 7.42 mm^−1^
*T* = 200 K
0.30 × 0.17 × 0.16 mm

#### Data collection

Bruker SMART 1000 CCD diffractometer
Absorption correction: multi-scan (*SADABS*; Bruker, 2000 ▶) *T*_min_ = 0.668, *T*_max_ = 1.000
4360 measured reflections
1524 independent reflections
1207 reflections with *I* > 2σ(*I*)
*R*_int_ = 0.043

#### Refinement

*R*[*F* ^2^ > 2σ(*F* ^2^)] = 0.033
*wR*(*F* ^2^) = 0.097
*S* = 1.14
1524 reflections
78 parameters
H-atom parameters constrained
Δρ_max_ = 0.92 e Å^−3^
Δρ_min_ = −0.76 e Å^−3^

### 

Data collection: *SMART* (Bruker, 2000 ▶); cell refinement: *SAINT* (Bruker, 2000 ▶); data reduction: *SAINT*; program(s) used to solve structure: *SHELXS97* (Sheldrick, 2008 ▶); program(s) used to refine structure: *SHELXL97* (Sheldrick, 2008 ▶); molecular graphics: *PLATON* (Spek, 2009 ▶); software used to prepare material for publication: *SHELXL97*.

## Supplementary Material

Crystal structure: contains datablock(s) global, I. DOI: 10.1107/S1600536811049919/pk2362sup1.cif

Structure factors: contains datablock(s) I. DOI: 10.1107/S1600536811049919/pk2362Isup2.hkl

Additional supplementary materials:  crystallographic information; 3D view; checkCIF report

## Figures and Tables

**Table:** 

Mn1—N1	2.300 (3)
Mn1—N2	2.322 (3)
Mn1—Br1	2.6094 (10)

**Table:** 

N1—Mn1—N2	71.21 (11)
Br1^i^—Mn1—Br1	97.93 (5)

Symmetry code: (i) 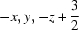.

**Table 2:** Hydrogen-bond geometry (Å, °)

*D*—H⋯*A*	*D*—H	H⋯*A*	*D*⋯*A*	*D*—H⋯*A*
O1—H1*A*⋯Br1^ii^	0.84	2.57	3.356 (3)	156
O1—H1*B*⋯Br1^iii^	0.84	2.61	3.394 (4)	157
C2—H2⋯O1^iv^	0.95	2.45	3.364 (5)	161

Symmetry codes: (ii) 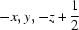; (iii) 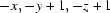; (iv) 
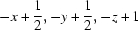
.

**Fig. 1.**
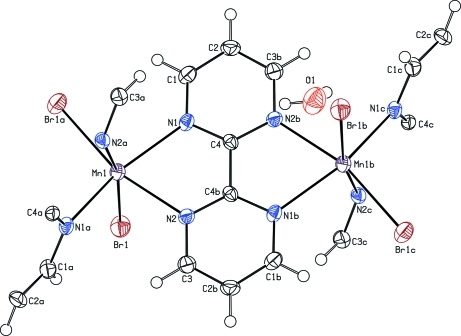
A fragment structure of the title compound, with displacement ellipsoids drawn at the 50% probability level for non-H atoms [symmetry codes: (a) -x, y, 3/2 - z, (b) -x, -y, 1 - z, (c) x, -y, -1/2 + z].

**Fig. 2.**
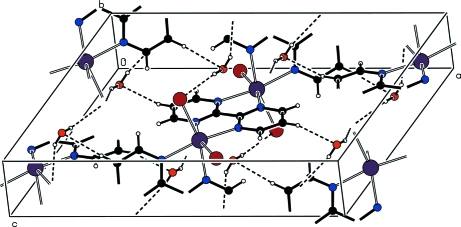
View of the unit-cell contents of the title compound. Hydrogen-bond interactions are drawn with dashed lines.

## supplementary crystallographic information

### Comment

The title compound, [MnBr_2_(bpym)]_n_.2nH_2_O (bpym = 2,2'-bipyrimidine, C_8_H_6_N_4_), consists of a neutral complex polymer and solvent water molecules. The compound is isomorphous with the chloro analogue [MnCl_2_(bpym)]_n_.2nH_2_O (Armentano *et al.*, 2003).

The asymmetric unit contains one half of a repeat unit of the polymer and a water molecule (Fig. 1). In the polymer, the symmetry related Mn^II^ ions are bridged by the bis(chelating) bpym ligands, thereby forming a chain structure along the *c* axis, and are six-coordinated in a distorted *cis*-N_4_Br_2_ octahedral environment by four N atoms of the two different bpym ligands and two bromide anions. The Br atoms are *cis* with respect to each other. The main contributions to the distortion are the tight N—Mn—N chelate angle and the Br—Br repelling (<N1—Mn1—N2 = 71.21 (11)° and <Br1—Mn1—Br1*^a^* = 97.93 (5)°; symmetry code a: -*x*, *y*, 3/2 - *z*), which result in non-linear *trans* axes (<N1—Mn1—N1*^a^* = 153.49 (16)° and <N2—Mn1—Br1*^a^* = 165.13 (7)°). The Mn—N bond lengths are almost equivalent (Table 1). In the crystal structure, the complex polymers and solvent water molecules are linked by intermolecular O—H···Br and C—H···O hydrogen bonds, forming a two-dimensional layer structure extending parallel to *ac* plane (Fig. 2, Table 2). The chains are stacked along the *b* axis.  The shortest ring centroid-centroid distance is 5.337 (3) Å.

### Experimental

To a solution of MnBr_2_.4H_2_O (0.2867 g, 1.000 mmol) in EtOH (30 ml) was added 2,2'-bipyrimidine (0.1584 g, 1.002 mmol) and stirred for 3 h at room temperature. The precipitate was separated by filtration, washed with EtOH and dried at 50 °C, to give a yellow powder (0.3441 g). Crystals suitable for X-ray analysis were obtained by slow evaporation from a methanol solution.

### Refinement

Carbon-bound H atoms were positioned geometrically and allowed to ride on their respective parent atoms [C—H = 0.95 Å and *U*_iso_(H) = 1.2*U*_eq_(C)]. The H atoms of the solvent water molecule were located in a difference Fourier map then allowed to ride on their parent O atom in the final cycles of refinement with O—H = 0.84 Å and *U*_iso_(H) = 1.5 *U*_eq_(O). The highest peak (0.92 e Å^-3^) and the deepest hole (-0.75 e Å^-3^) in the difference Fourier map are located 1.26 Å and 0.96 Å from the atoms H1B and Br1, respectively.

### Crystal data

**Table:**

[MnBr_2_(C_8_H_6_N_4_)]·2H_2_O	*F*(000) = 788
*M**_r_* = 408.96	*D*_x_ = 2.165 Mg m^−^^3^
Monoclinic, *C*2/*c*	Mo *K*α radiation, λ = 0.71073 Å
Hall symbol: -C 2yc	Cell parameters from 2416 reflections
*a* = 17.950 (9) Å	θ = 2.4–28.3°
*b* = 8.263 (4) Å	µ = 7.42 mm^−^^1^
*c* = 10.188 (5) Å	*T* = 200 K
β = 123.888 (8)°	Stick, yellow
*V* = 1254.4 (10) Å^3^	0.30 × 0.17 × 0.16 mm
*Z* = 4	

### Data collection

**Table:**

Bruker SMART 1000 CCD diffractometer	1524 independent reflections
Radiation source: fine-focus sealed tube	1207 reflections with *I* > 2σ(*I*)
graphite	*R*_int_ = 0.043
φ and ω scans	θ_max_ = 28.3°, θ_min_ = 2.7°
Absorption correction: multi-scan (*SADABS*; Bruker, 2000)	*h* = −23→23
*T*_min_ = 0.668, *T*_max_ = 1.000	*k* = −11→8
4360 measured reflections	*l* = −13→12

### Refinement

**Table:**

Refinement on *F*^2^	Primary atom site location: structure-invariant direct methods
Least-squares matrix: full	Secondary atom site location: difference Fourier map
*R*[*F*^2^ > 2σ(*F*^2^)] = 0.033	Hydrogen site location: inferred from neighbouring sites
*wR*(*F*^2^) = 0.097	H-atom parameters constrained
*S* = 1.14	*w* = 1/[σ^2^(*F*_o_^2^) + (0.0395*P*)^2^] where *P* = (*F*_o_^2^ + 2*F*_c_^2^)/3
1524 reflections	(Δ/σ)_max_ < 0.001
78 parameters	Δρ_max_ = 0.92 e Å^−^^3^
0 restraints	Δρ_min_ = −0.76 e Å^−^^3^

### Special details

**Table:**

Geometry. All e.s.d.'s (except the e.s.d. in the dihedral angle between two l.s. planes) are estimated using the full covariance matrix. The cell e.s.d.'s are taken into account individually in the estimation of e.s.d.'s in distances, angles and torsion angles; correlations between e.s.d.'s in cell parameters are only used when they are defined by crystal symmetry. An approximate (isotropic) treatment of cell e.s.d.'s is used for estimating e.s.d.'s involving l.s. planes.
Refinement. Refinement of *F*^2^ against ALL reflections. The weighted *R*-factor *wR* and goodness of fit *S* are based on *F*^2^, conventional *R*-factors *R* are based on *F*, with *F* set to zero for negative *F*^2^. The threshold expression of *F*^2^ > σ(*F*^2^) is used only for calculating *R*-factors(gt) *etc*. and is not relevant to the choice of reflections for refinement. *R*-factors based on *F*^2^ are statistically about twice as large as those based on *F*, and *R*- factors based on ALL data will be even larger.

### Fractional atomic coordinates and isotropic or equivalent isotropic displacement parameters (Å^2^)

**Table:**

	*x*	*y*	*z*	*U*_iso_*/*U*_eq_	
Mn1	0.0000	0.20755 (10)	0.7500	0.0207 (2)	
Br1	−0.11908 (3)	0.41487 (5)	0.54940 (5)	0.03063 (17)	
N1	0.07975 (19)	0.1437 (4)	0.6421 (3)	0.0197 (6)	
N2	−0.07926 (19)	0.0143 (4)	0.5541 (3)	0.0200 (6)	
C1	0.1597 (2)	0.2064 (5)	0.6848 (4)	0.0243 (8)	
H1	0.1869	0.2857	0.7660	0.029*	
C2	0.2028 (2)	0.1594 (5)	0.6146 (4)	0.0272 (9)	
H2	0.2598	0.2025	0.6478	0.033*	
C3	−0.1606 (2)	−0.0473 (5)	0.5058 (5)	0.0254 (8)	
H3	−0.1892	−0.0125	0.5561	0.030*	
C4	0.0437 (2)	0.0356 (4)	0.5240 (4)	0.0177 (7)	
O1	0.0770 (2)	0.2654 (4)	0.2093 (4)	0.0470 (8)	
H1A	0.0833	0.3297	0.1523	0.070*	
H1B	0.0717	0.3369	0.2623	0.070*	

### Atomic displacement parameters (Å^2^)

**Table:**

	*U*^11^	*U*^22^	*U*^33^	*U*^12^	*U*^13^	*U*^23^
Mn1	0.0210 (4)	0.0249 (5)	0.0165 (4)	0.000	0.0106 (3)	0.000
Br1	0.0324 (3)	0.0312 (3)	0.0244 (3)	0.00758 (15)	0.0134 (2)	0.00491 (15)
N1	0.0224 (15)	0.0209 (17)	0.0148 (15)	−0.0028 (12)	0.0097 (13)	−0.0009 (12)
N2	0.0187 (14)	0.0245 (18)	0.0166 (15)	−0.0033 (12)	0.0097 (13)	−0.0003 (12)
C1	0.0251 (19)	0.027 (2)	0.0189 (19)	−0.0074 (15)	0.0110 (16)	−0.0016 (14)
C2	0.0195 (18)	0.039 (3)	0.025 (2)	−0.0068 (16)	0.0131 (17)	−0.0006 (17)
C3	0.0231 (19)	0.032 (2)	0.022 (2)	−0.0018 (16)	0.0130 (17)	0.0006 (16)
C4	0.0180 (17)	0.0193 (19)	0.0172 (17)	0.0038 (13)	0.0107 (15)	0.0028 (13)
O1	0.058 (2)	0.045 (2)	0.041 (2)	−0.0146 (16)	0.0301 (18)	−0.0070 (15)

### Geometric parameters (Å, °)

**Table:**

Mn1—N1	2.300 (3)	C1—C2	1.371 (5)
Mn1—N1^i^	2.300 (3)	C1—H1	0.9500
Mn1—N2	2.322 (3)	C2—C3^ii^	1.379 (5)
Mn1—N2^i^	2.322 (3)	C2—H2	0.9500
Mn1—Br1^i^	2.6094 (10)	C3—C2^ii^	1.379 (5)
Mn1—Br1	2.6094 (10)	C3—H3	0.9500
N1—C4	1.340 (5)	C4—N2^ii^	1.333 (4)
N1—C1	1.349 (4)	C4—C4^ii^	1.481 (7)
N2—C4^ii^	1.333 (4)	O1—H1A	0.8400
N2—C3	1.353 (5)	O1—H1B	0.8400
			
N1—Mn1—N1^i^	153.49 (16)	C1—N1—Mn1	126.2 (2)
N1—Mn1—N2	71.21 (11)	C4^ii^—N2—C3	116.4 (3)
N1^i^—Mn1—N2	90.38 (11)	C4^ii^—N2—Mn1	117.3 (2)
N1—Mn1—N2^i^	90.38 (11)	C3—N2—Mn1	126.2 (2)
N1^i^—Mn1—N2^i^	71.21 (11)	N1—C1—C2	122.1 (4)
N2—Mn1—N2^i^	93.08 (16)	N1—C1—H1	119.0
N1—Mn1—Br1^i^	93.93 (8)	C2—C1—H1	119.0
N1^i^—Mn1—Br1^i^	103.45 (8)	C1—C2—C3^ii^	117.7 (3)
N2—Mn1—Br1^i^	165.13 (7)	C1—C2—H2	121.2
N2^i^—Mn1—Br1^i^	86.37 (8)	C3^ii^—C2—H2	121.2
N1—Mn1—Br1	103.45 (8)	N2—C3—C2^ii^	121.5 (3)
N1^i^—Mn1—Br1	93.93 (8)	N2—C3—H3	119.2
N2—Mn1—Br1	86.37 (8)	C2^ii^—C3—H3	119.2
N2^i^—Mn1—Br1	165.13 (7)	N2^ii^—C4—N1	126.2 (3)
Br1^i^—Mn1—Br1	97.93 (5)	N2^ii^—C4—C4^ii^	116.9 (4)
C4—N1—C1	116.1 (3)	N1—C4—C4^ii^	116.9 (4)
C4—N1—Mn1	117.7 (2)	H1A—O1—H1B	96.1
			
N1^i^—Mn1—N1—C4	49.5 (3)	N1—Mn1—N2—C3	−177.7 (3)
N2—Mn1—N1—C4	1.3 (3)	N1^i^—Mn1—N2—C3	21.8 (3)
N2^i^—Mn1—N1—C4	94.4 (3)	N2^i^—Mn1—N2—C3	93.0 (3)
Br1^i^—Mn1—N1—C4	−179.2 (3)	Br1^i^—Mn1—N2—C3	−179.6 (2)
Br1—Mn1—N1—C4	−80.1 (3)	Br1—Mn1—N2—C3	−72.1 (3)
N1^i^—Mn1—N1—C1	−131.5 (3)	C4—N1—C1—C2	−1.7 (6)
N2—Mn1—N1—C1	−179.7 (3)	Mn1—N1—C1—C2	179.2 (3)
N2^i^—Mn1—N1—C1	−86.5 (3)	N1—C1—C2—C3^ii^	1.7 (6)
Br1^i^—Mn1—N1—C1	−0.2 (3)	C4^ii^—N2—C3—C2^ii^	1.9 (5)
Br1—Mn1—N1—C1	99.0 (3)	Mn1—N2—C3—C2^ii^	178.5 (3)
N1—Mn1—N2—C4^ii^	−1.1 (2)	C1—N1—C4—N2^ii^	−0.2 (6)
N1^i^—Mn1—N2—C4^ii^	−161.7 (3)	Mn1—N1—C4—N2^ii^	179.0 (3)
N2^i^—Mn1—N2—C4^ii^	−90.5 (3)	C1—N1—C4—C4^ii^	179.5 (4)
Br1^i^—Mn1—N2—C4^ii^	−3.0 (5)	Mn1—N1—C4—C4^ii^	−1.3 (5)
Br1—Mn1—N2—C4^ii^	104.4 (3)		

Symmetry codes: (i) −*x*, *y*, −*z*+3/2; (ii) −*x*, −*y*, −*z*+1.

### Hydrogen-bond geometry (Å, °)

**Table:**

*D*—H···*A*	*D*—H	H···*A*	*D*···*A*	*D*—H···*A*
O1—H1A···Br1^iii^	0.84	2.57	3.356 (3)	156.
O1—H1B···Br1^iv^	0.84	2.61	3.394 (4)	157.
C2—H2···O1^v^	0.95	2.45	3.364 (5)	161.

Symmetry codes: (iii) −*x*, *y*, −*z*+1/2; (iv) −*x*, −*y*+1, −*z*+1; (v) −*x*+1/2, −*y*+1/2, −*z*+1.
